# Estimation of Ankle Joint Power during Walking Using Two Inertial Sensors

**DOI:** 10.3390/s19122796

**Published:** 2019-06-21

**Authors:** Xianta Jiang, Mohsen Gholami, Mahta Khoshnam, Janice J. Eng, Carlo Menon

**Affiliations:** 1 Menrva Research Group, Schools of Mechatronic Systems & Engineering Science, Simon Fraser University, 8888 University Dr, Burnaby, BC V5A 1S6, Canada; xiantaj@sfu.ca (X.J.); mohsen_gholami@sfu.ca (M.G.); mahta_khoshnam_tehrani@sfu.ca (M.K.); 2 Department of Physical Therapy, GF Strong Rehab Centre, Vancouver Coastal Health Research Institute, Vancouver Campus, University of British Columbia and Rehabilitation Research Program, 212–2177 Wesbrook Mall, Vancouver, BC V6T 1Z3, Canada; janice.eng@ubc.ca

**Keywords:** IMU, joint power, ankle power, gait analysis

## Abstract

(1) Background: Ankle joint power, as an indicator of the ability to control lower limbs, is of great relevance for clinical diagnosis of gait impairment and control of lower limb prosthesis. However, the majority of available techniques for estimating joint power are based on inverse dynamics methods, which require performing a biomechanical analysis of the foot and using a highly instrumented environment to tune the parameters of the resulting biomechanical model. Such techniques are not generally applicable to real-world scenarios in which gait monitoring outside of the clinical setting is desired. This paper proposes a viable alternative to such techniques by using machine learning algorithms to estimate ankle joint power from data collected by two miniature inertial measurement units (IMUs) on the foot and shank, (2) Methods: Nine participants walked on a force-plate-instrumented treadmill wearing two IMUs. The data from the IMUs were processed to train and test a random forest model to estimate ankle joint power. The performance of the model was then evaluated by comparing the estimated power values to the reference values provided by the motion tracking system and the force-plate-instrumented treadmill. (3) Results: The proposed method achieved a high accuracy with the correlation coefficient, root mean square error, and normalized root mean square error of 0.98, 0.06 w/kg, and 1.05% in the intra-subject test, and 0.92, 0.13 w/kg, and 2.37% in inter-subject test, respectively. The difference between the predicted and true peak power values was 0.01 w/kg and 0.14 w/kg with a delay of 0.4% and 0.4% of gait cycle duration for the intra- and inter-subject testing, respectively. (4) Conclusions: The results of this study demonstrate the feasibility of using only two IMUs to estimate ankle joint power. The proposed technique provides a basis for developing a portable and compact gait monitoring system that can potentially offer monitoring and reporting on ankle joint power in real-time during activities of daily living.

## 1. Introduction

Walking, the most basic means of transportation, is one of the most popular and important physical human activities in daily living. Gait analysis refers to methods that quantify different features of walking motion and help identify individual movement patterns. Studying the gait parameters, such as speed, step length, cadence, gait phases, and step variation [1,2], can help analyze performance and efficiency in sports activities, such as running [3], and is also a useful tool for understanding the gait abnormalities that might occur because of neurological, orthopedic, or medical conditions [4]. Clinical monitoring of gait is one of the common methods to diagnose and characterize disorders that might lead to falls and subsequent injuries [5,6,7,8,9]. Among various gait parameters, ankle joint power, as an indicator of the ability to control lower limbs, is of great relevance in clinical applications. Together with knee and hip joints and muscles power, positive ankle joint power is mainly generated at the end of a stance phase right before the foot breaks the ground, which accelerates the leg into swing phase, and redirects and pushes the body’s center of mass forward [10,11]. It has been reported that a decrease in ankle and knee power results in shorter step length in the elderly [12]. In individuals with ankle osteoarthritis, smaller ankle power is accompanied by reduced cadence, lower walking speed, shorter strides, and increased step times [13]. It is also reported that in stroke-affected individuals who demonstrate hemiparetic gait, the unaffected side compensates for the affected side by performing the majority of work and power during walking, and, although the ankle, knee, and hip power profile looks similar for both sides, the power amplitude is lower for the affected side [14,15]. These evidences suggest that ankle joint power is one of the gait indices to be considered to diagnose and characterize the gait deficits and can be used to develop rehabilitation protocols to improve walking performance [14]. Detecting the timing of peak ankle joint power can also help generate the timing sequence for lower limb prosthesis [16].

Estimating ankle joint power in lab settings is commonly based on inverse dynamic analysis, in which a biomechanical model of the ankle is needed to read the data from a force-plate-instrumented treadmill and an optical motion tracking system and output ankle joint power. However, regardless of the characteristics of the biomechanical model, a setup consisting of optical trackers and force-plate-instrumented treadmill is not a universal solution. Applications that require real-time monitoring of joint power in out-of-clinic situations, such as prosthesis control or in-home gait monitoring scenarios, call for a portable and user-friendly device.

Inertial measurement units (IMUs) have recently received increasing attention in systems designed to track limb motions. Their low cost, low power consumption, compact size, and portability are among the features that make IMUs appealing for such an application. It has been demonstrated that a system consisting of a set of IMUs, mounted on full-body segments to capture limb kinematics and force plates to record ground reaction force, can provide the information required for calculating joint powers [17]. More specifically, three IMUs were mounted on the pelvis and lower legs to estimate the knee joint angle and vertical ground reaction force. The estimated joint angles were then compared with values obtained from two reference systems, respectively, where one consisted of an inertial full-body motion tracking system with 17 IMUs and force-plate-instrumented treadmill, and another one was an optical motion tracking system plus a force-plate-instrumented treadmill. However, considering the suggested placement for IMUs and that they are worn on three different limbs, it is still not as convenient as using only two IMUs. Biosignals have also been used to estimate joint power by monitoring the activity of muscles surrounding the ankle joint. For instance, it was shown that ankle joint power can be obtained by multiplying the ankle muscle force by the contraction velocity. The muscle force was estimated using the electromyography (EMG) signals collected with 12 intramuscular electrodes [18]. However, this method is intrusive and non-portable, and also requires extensive calibration, and, therefore, does not suit general walking sessions performed outside of the clinical setting.

The work presented in this paper explores the feasibility of developing a compact, wearable, and real-time ankle joint power monitoring system that can potentially be used outside of clinic. We hypothesized that ankle joint power could be estimated with good accuracy (correlation coefficient > 0.9) using data from two IMUs: one mounted on the foot and the other on the shank.

## 2. Materials and Methods

### 2.1. Experimental Setup

**Figure 1:**
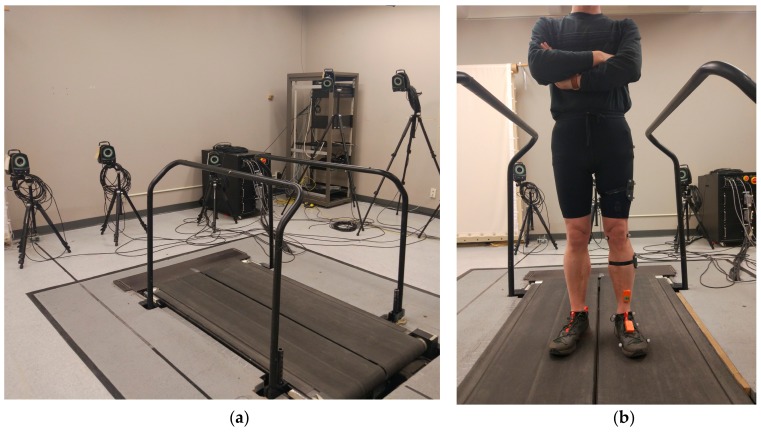
The experimental setup. (**a**) The force-plate-instrumented treadmill with the motion tracking system recorded movements of lower limbs and (**b**) motion tracker markers and Inertial measurement units (IMUs) mounted on left lower limb.

Two wireless IMU motion trackers (MTw Awinda, Xsens, Enschede, The Netherlands) were mounted on the shank and foot of each participant for measuring the acceleration and angular velocity of each segment during the walking trial (Figure 1). A Vicon motion capture system (Vicon, Oxford, UK), consisting of seven infrared motion tracking cameras and 13 reflective markers with diameter of 14 mm, which were mounted on participants’ shank and foot, was used to record the kinematics of walking. To capture ground reaction force as well as the free moment during the walking trial, a Bertec split belt treadmill (Bertec Corporation, Columbus, OH, USA) equipped with force plates was used. With such a setup, the data from the treadmill could be used together with the data from the motion tracking system to produce the reference values of ankle joint power through a biomechanical model, which was built from the static trial data recorded at the beginning of trials for each participant (see Section 2.2). The signals from the IMUs were synchronized with the reference signals from the motion tracker and the treadmill, by sending two synchronization impulse signals from both the IMUs and the motion tracker system to the treadmill. The data from all three components were recorded at 100 Hz sample rate.

### 2.2. Protocol and Procedure

Nine young healthy adult participants were recruited for this study, all males. The average height and weight of the participants were 176 ± 7 cm, 72 ± 9 kg, respectively. The study protocol was approved by the Office of Research Ethics at Simon Fraser University, and all participants provided informed consent. A static trial was arranged before starting the walking trials during which a same set of 13 markers were used. The foot segment was defined by two proximal markers (the medial and lateral ankle malleoli) and two distal markers (first and fifth metatarsal head markers). Three other markers were also used as the tracking markers for foot segment, which were placed on the back and side of the shoes. The shank segment was defined by two proximal markers (medial and lateral femoral condyles) and two distal markers (medial and lateral malleoli). Four markers mounted on a plate were also used as the tracking markers of the shank. Six static markers (medial/lateral femoral condyles, medial/lateral malleoli, and first and fifth metatarsal heads) were removed after the static trial. The seven remaining markers were the four markers on the shank and the three markers on the foot, which were used for data collection during the walking trials. To ensure that the performance of the motion capture system was not compromised by lights reflected from the environment, all possible reflective materials on participants’ clothes and shoes and in the environment seen by the cameras were removed or covered by non-reflective tape. The participants walked on the treadmill at five different speeds, namely 0.4 m/s, 0.7 m/s, 1.0 m/s, 1.3 m/s, and 1.6 m/s, each for 1 minute, in order from low to high speed. These values were chosen from the normal range of walking speeds for an adult human such that they included both lower and higher speeds [19]. We did not randomize the speed order because it was easier for the participant to anticipate the next speed to walk. At each walking speed, the data collection was started after the treadmill reached the target speed for 1 minute after the initial acceleration period. The participants were allowed to practice walking on the treadmill to get warmed up. Between each two consecutive trials, the experimenter asked the participant whether he needed a rest. All data collected with the IMUs, the treadmill, and the motion tracking system were saved to be processed later.

### 2.3. Data Analysis

Ankle joint power is defined as the multiplication of the ankle joint angular velocity and joint moment. In this study, determining the orientation of the foot segment, i.e., the ankle joint, was made possible by the strategic placement of IMUs. The foot and the shank IMUs both moved according to the ankle movement, and, therefore, the difference between their signals correlated with the angle of the ankle joint from which the angular velocity could be obtained. The other contributor to joint power, i.e., the joint moment, was calculated from the acceleration information provided by the IMUs. It should be noted that, in this study, there was no need to explicitly subtract one IMU signal from the other to obtain the ankle angle. The signals from the two IMUs were simply concatenated and fed into the machine learning algorithm which subsequently estimated the angular velocity. This section provides more details on how data from different sources were used.

#### 2.3.1. Reference Ankle Joint Power

For each subject and at each speed, the sagittal ankle joint power was calculated by the built-in module in Visual3D using the data recorded by the motion tracking system and ground reaction force and moment data recorded by the treadmill. Following the method recommended in the literature, all marker trajectories data were smoothed using a 4th order Butterworth low-pass filter, with a cut-off frequency of 6 Hz [18].

#### 2.3.2. Data Processing

The data collected by IMUs were smoothed by a median filter using a 5-sample window. Then the data went through a feature extraction procedure using a 110-ms window. Signal features considered at this stage included root-mean-square (RMS), sum of absolute value (SAV), mean absolute deviation (MAV), variance (VAR), wave length (WL), slope sign changes (SSC), and simple square integral (SSI), mean wavelet with db7 (db7), difference absolute standard deviation value (DASDV), average amplitude change (AAC), log detector (LD), linear fit (LF), and parabolic fit (PF) [20,21], which were also proved to be effective for gait phase partitioning [1]. These extracted features were further normalized to a uniformed range between 0 and 1.

Random forest learning algorithm was employed to train and test the ankle power prediction model. Random forests are an ensemble of prediction trees in which each tree is trained based on a random selection of part of the training dataset and usually achieve a high accuracy by voting for the best result from those produced by the trees [22,23]. The random forest regressor algorithm is available in Statistics and Machine Learning Toolbox in MATLAB R2019a (The MathWorks, Inc., Natick, MA, USA). The number of trees in each iteration was set to 50, and the leaf number of each tree was set to a default value of 5.

Preliminary results of training the abovementioned random forest model with the data collected by IMUs showed that, in the majority of cases, the obtained model underestimated the amplitude of ankle joint power at the peaks. To resolve this issue, a quantile regression algorithm was implemented, and, subsequently, a specific quantile probability was chosen for each response. The quantile regression algorithm generates a set of possible candidate responses for each input data and takes the median of these candidates as the default value. To compensate for the power underestimation, the algorithm can be modified to choose a different quantile possibility for the response. For example, a higher quantile possibility should be assigned to the peak while the valley points should receive lower quantile possibility values. The quantile possibility was determined by the normalized median quantile possibility value of the corresponding predicted candidates. The first half of the testing data at each speed for each subject was used to calculate the maximum and minimum power values. These two parameters were then used to normalize the second half of testing data to the range of [0 1] using the following equation:

(1) $$X\_norm=\frac{X\_2nd-\min\left(X\_1st\right)}{\max\left(X\_1st\right)-\min\left(X\_1st\right)},$$

where X_norm is the normalized data, and X_1st and X_2nd represent the first and second half of original data, respectively. Once the normalized value is derived, for example, X_norm = 0.75, then the 75% percentile of the candidate value is chosen.

#### 2.3.3. Evaluation

**Figure 2:**
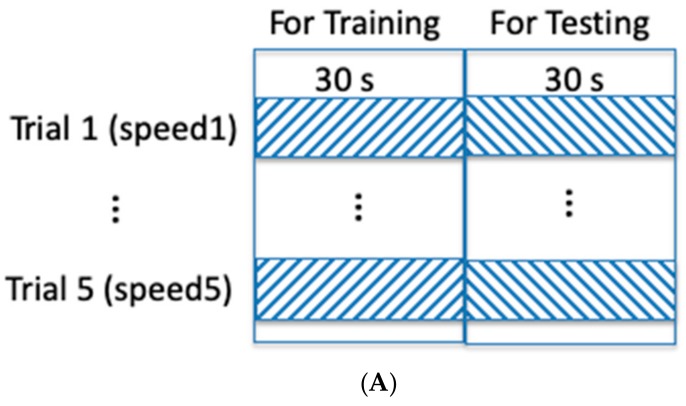

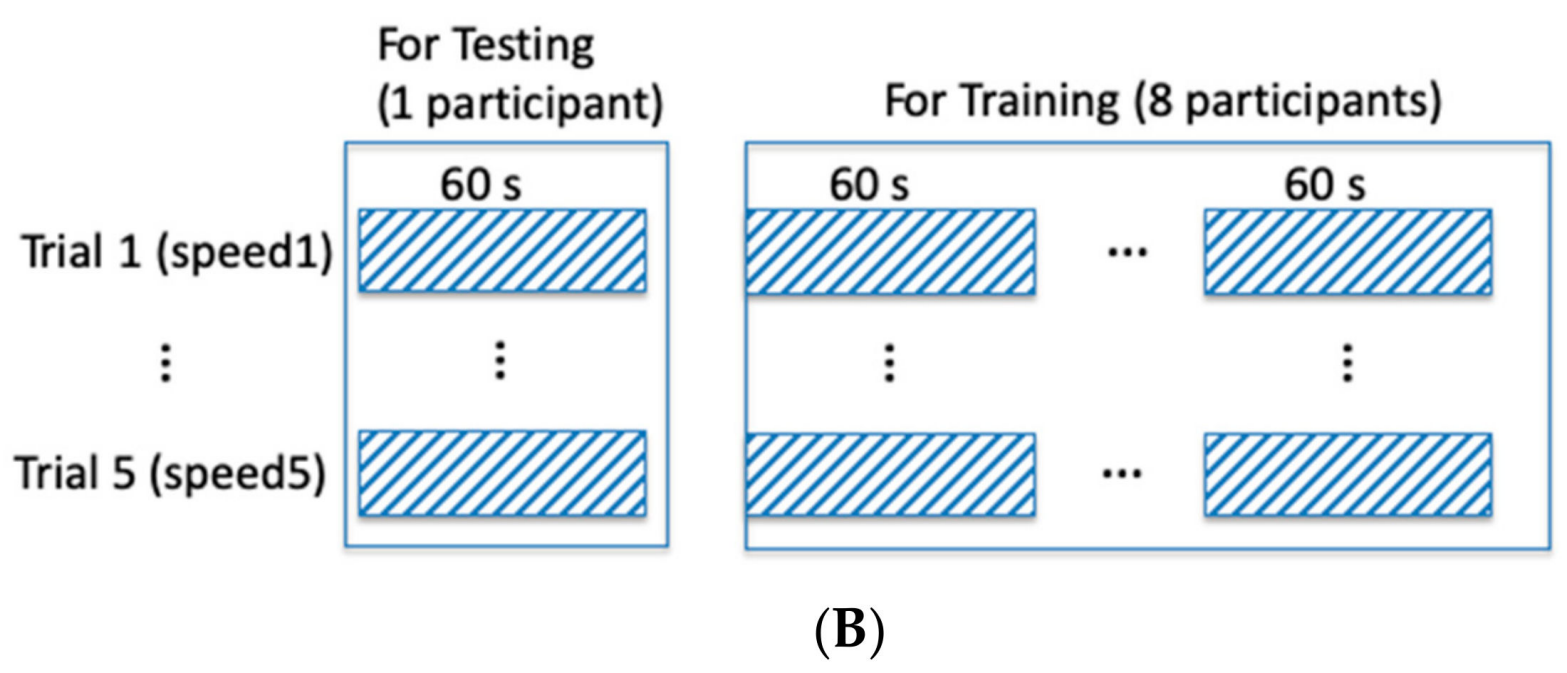
The schematic drawings of intra-subject and inter-subject testing. (**A**) The intra-subject testing: the first half data of each trial (speed) of a subject are used for training a IMU-to-power model, and tested with the rest half data from the same subject. (**B**) The inter-subject testing: IMU data at all five speeds from one of the nine participants were chosen as testing data, and IMU data from the remaining eight participants at all five speeds were assigned to the training dataset. This process was repeated until the data from each participant were used as testing data.

The performance of the sagittal ankle joint power estimation method was quantitatively assessed by comparing the obtained power values to the reference ones calculated by Visual3D using the force data recorded from the force-plate-instrumented treadmill and the kinematic data from the motion tracking system. To validate the implemented machine learning algorithm for joint power estimation, both intra-subject and inter-subject validations were employed. In intra-subject evaluation, the training and testing data were from the same subject, and a single IMU-to-power model was trained for each subject. More specifically, the first half of the IMU data from each subject was used to train a subject-dependent model, and the second half of the data from the same subject was used for testing (Figure 2A). In inter-subject evaluation, IMU data at all five speeds from one of the nine participants were chosen as the testing data, and IMU data from the remaining eight participants at all five speeds were assigned to the training dataset. This process was repeated until the data from each participant were used as testing data (Figure 2B). It is worthwhile noting that the inter-subject evaluation adds to the practicality of the system, since a wearable device, designed based on the proposed method, should contain a pre-trained model before delivery to the user. This is an essential feature as, in practical scenarios, the end users generally do not have access to a motion tracker or a force-plate-instrumented treadmill to obtain reference data required to train the model. In both evaluations, the accuracy was then calculated by averaging the accuracies obtained across all of the subjects, at all considered walking speeds. As explained in Section 2.3.2, the first half of each data signal was used to derive the minimum to maximum range for the normalization in compensation for the underestimation of the power amplitude (Equation (1)). Consequently, only the second half of the testing data of each trial was included in the inter-subject.

The correlation coefficient (R), the root mean squared error (RMSE), and the normalized root mean squared error (NRMSE) were considered as outcome measures to evaluate the model performance. R is a statistical measure that quantifies the similarity between an estimated value and its reference. The peak value of the power and its occurrence position in each gait cycle were also reported. As the peak value is determined within each gait cycle, we defined the start of a gait cycle by ground reaction force going above 0 N. The ground reaction force was recorded by the force plate in the treadmill.

## 3. Results

**Figure 3:**
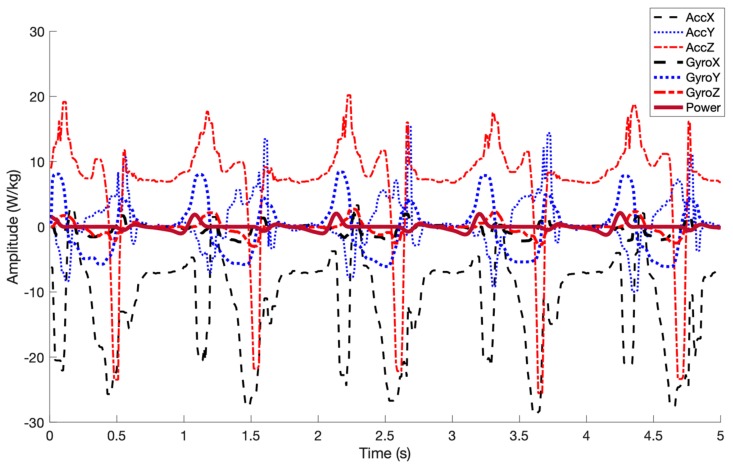
Sample of collected IMU raw signals for a segment, including four gait cycles, overlaid with reference ankle joint power labels (bold purple line). The amplitude of ankle joint power is normalized by body weight in watts/kg.

**Figure 4:**
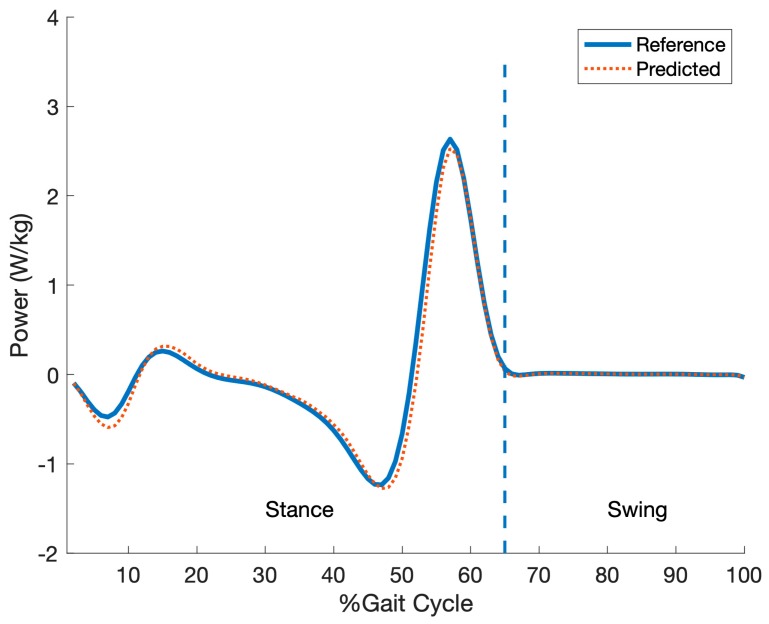
An example of the predicted ankle joint power compared to the reference power for a subject walking at a speed of 1.3 m/s. Solid and dot curves represent the reference and the predicted ankle joint power profiles, respectively. The amplitude of the ankle joint power is normalized by body weight in watts/kg.

Each participant performed five trials (one trial at each considered speed). Considering the sampling rate (100 Hz) and the 1-minute duration of each of the 5 trials, 30,000 IMU data points were collected from each subject, resulting in a total of 270,000 samples collected across 45 recorded trials. After an initial examination of the data, eight trials were excluded (18% of data) because, during these trials, motion capture markers were missed or the ground reaction force was not correctly recorded while the participants walked on only one of the belts. A sample of collected IMU signals and the corresponding reference ankle joint power in the gait cycles is shown in Figure 3. An example of ankle joint power profile for a subject at a walking speed 1.3 m/s is shown in Figure 4.

### 3.1. Intra-Subject Test Accuracy

The regression model achieved an intra-subject regression accuracy of R = 0.98 ± 0.01, RMSE = 0.06 ± 0.01 w/kg, and NRMSE = 1.05% ± 0.13%. The accuracies per speed are listed in Table 1.

The mean predicted and true peak joint power across five speeds and all subjects are 1.69 w/kg and 1.70 w/kg, respectively, with their occurrence positions at 58.4% and 58.0% of the gait cycle duration. The relative error of peak power, i.e., (predicted power value – true power value)/power value range, is -0.6%. Details of peak power and its occurrence per speed are listed in Table 2.

**Figure 5:**
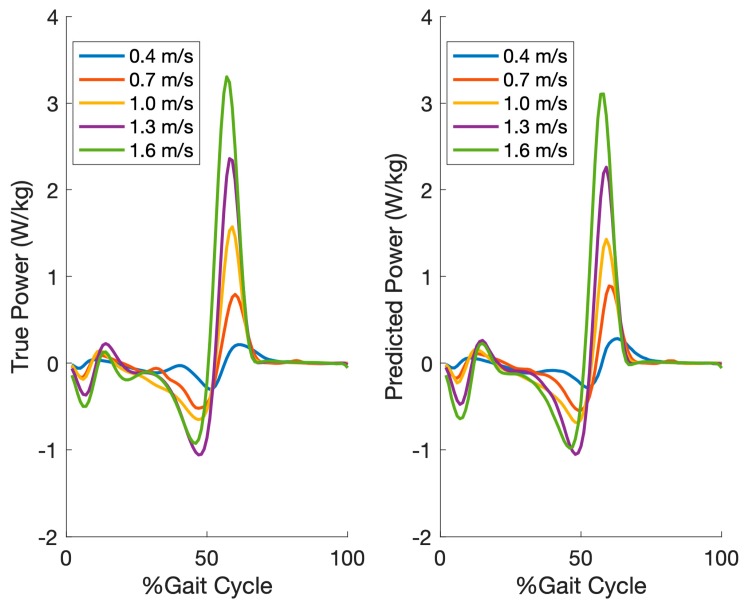
The averaged reference and predicted (intra-subject) ankle joint power across all gait cycles of each participant per speed for one of the participants. The amplitude of ankle joint power is normalized by body weight in watts/kg.

Figure 5 shows the reference and predicted (intra-subject) ankle joint power averaged across all gait cycles of each participant per speed for one of the participants. 

### 3.2. Inter-Subject Test Accuracy

The regression model achieved a regression accuracy of R = 0.92 ± 0.04, RMSE = 0.13 ± 0.04 (w/kg), and NRMSE = 2.37% ± 0.60%. The accuracies per speed are listed in Table 3.

The mean predicted and true peak joint power across five speeds and all subjects are 1.84 w/kg and 1.70 w/kg, respectively, with their occurrence positions at 58.4% and 58.0% with respect to the gait cycle duration. The relative error of peak power, i.e., (predicted power–true power)/power range, is 6.8%. Details of peak power and its occurrence per speed are listed in Table 2.

**Figure 6:**
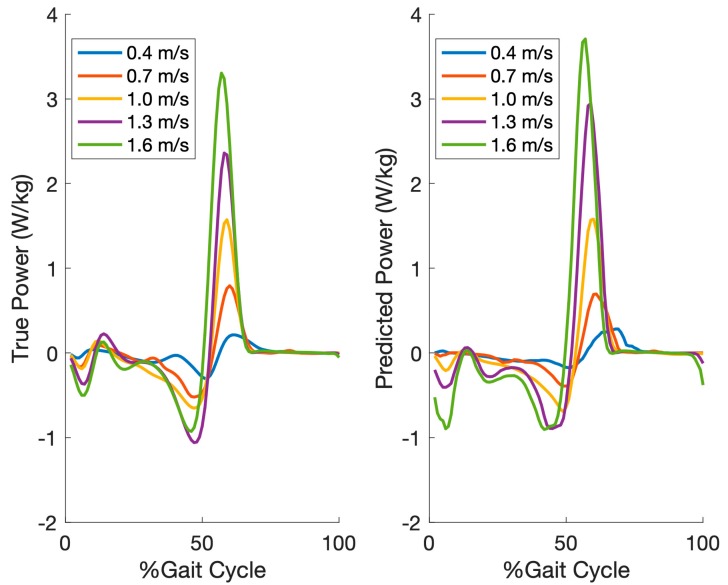
The averaged reference and predicted (inter-subject) ankle joint power across all gait cycles of each participant per speed for the same participant in Figure 5. The amplitude of ankle joint power is normalized by body weight in watts/kg.

Figure 6 shows the reference and predicted (inter-subject) ankle joint power averaged across all gait cycles of each participant per speed for the same participant in Figure 5.

## 4. Discussion

To the best of authors’ knowledge, this is the first study that uses only two IMUs to estimate ankle joint power. The obtained accuracy of R = 0.98 across all five speeds for the intra-subject is acceptable and comparable to that obtained using intramuscular electromyography (EMG) [18]. The practicality of the system was further assessed through inter-subject testing. Although the accuracy of the inter-subject test was lower than that of the intra-subject test (R = 0.92 versus R = 0.98), such an accuracy is still acceptable and also comparable to that reported in [17], which estimates vertical ground reaction forces using three IMUs with an NRMSE of 2.37% for the inter-subject test. Good accuracy in inter-subject testing is a desirable feature with practical benefits for general walking scenarios since the device can be pre-trained using the data collected from a sample group of individuals and delivered to general population without requiring further or lengthy tuning. However, this study considered only gait patterns of males within a limited range of heights, weights and ages, which might have boosted the model prediction accuracy. Therefore, a larger group of participants with demographic diversity should be sought for future studies.

The peak power value in gait cycles was also accurately estimated in intra-subject testing with an average error of 0.01 w/kg, which represents about 0.6% of the power value range from valley to peak. However, the error in estimating the peak power is about 0.14 w/kg in inter-subject testing, which is about 6.8% of the power value range. A possible explanation for this result is that the amplitude of power varies largely between individuals. Interestingly, regardless of the individual differences in terms of amplitude of the push-up power estimation, the occurrence of peak power in gait cycles was correctly detected in both intra- and inter-subject testing, both with a negligible average delay of 0.4%.

## 5. Limitations and Future Work

This study was conducted in a lab setting using an optical motion tracking system and a force-plate-instrumented treadmill for the purpose of model validation. Future work will explore walking outdoors using an inertial motion capture system for kinematic and insole pressure sensors for kinetic references. Walking at higher speeds and running scenarios will also be considered in the next steps of this study.

## 6. Conclusions

This paper presented a method that can estimate ankle joint power using only two IMUs, and can, thus, be applied to outdoor situations. Nine participants were asked to wear two IMUs on their foot and shank and to walk on a treadmill at different speeds. A random forests regressor was employed to map the IMUs signals to ankle joint power values. It was shown that the method achieves an accuracy of correlation coefficient R= 0.98 averaged from all studied speeds and NRMSE = 1.09%. The occurrence of peak power was correctly detected with a negligible delay of 0.4% of gait cycle duration in both intra- and inter-subject tastings. The results of this study suggest the feasibility of using miniature IMUs to estimate ankle joint power during walking.

## Figures and Tables

**Table 1:** Mean intra-subject testing accuracy obtained from each of the five walking speeds across all subjects.

	Speeds (m/s)				
Accuracy	0.4	0.7	1.0	1.3	1.7
Correlation coefficient (R)	0.94	0.97	0.98	0.98	0.98
Root mean squared error (RMSE)	0.03	0.04	0.06	0.08	0.10
Normalized root mean squared error (NRMSE)	0.49%	0.77%	1.00%	1.37%	1.71%

**Table 2:** Mean peak joint power of each gait cycle obtained from each of the five walking speeds across all subjects. Peak occurrence is reported as a percentage of the gait cycle duration.

	Speeds (m/s)				
	0.4	0.7	1.0	1.3	1.7
True Peak Power (w/kg)	0.34	0.91	1.71	2.52	3.04
Power Range (w/kg)	0.67	1.55	2.55	3.52	3.97
Predicted Peak Power (w/kg) (Intra-subject)	0.34	0.90	1.65	2.53	3.03
Predicted Peak Power (w/kg) (Inter-subject)	0.42	1.00	1.90	2.70	3.17
True Peak Occurrence	60.7%	60.3%	58.0%	56.6%	54.8%
Predicted Peak Occurrence (Intra-subject)	61.3%	60.4%	58.1%	57.1%	55.2%
Predicted Peak Occurrence (Inter-subject)	62.0%	60.5%	58.0%	56.8%	54.8%

**Table 3:** Mean inter-subject testing accuracy obtained for each of the five walking speeds across all subjects.

	Speeds (m/s)				
Accuracy	0.4	0.7	1.0	1.3	1.7
Correlation coefficient (R)	0.84	0.87	0.91	0.92	0.93
Root mean squared error (RMSE)	0.06	0.11	0.14	0.17	0.21
Normalized root mean squared error (NRMSE)	1.0%	1.8%	2.5%	3.0%	3.8%
